# Diagnostic performance of plasma p-Tau217, p-Tau181, and p-Tau231 across the Alzheimer’s disease continuum: a network meta-analysis

**DOI:** 10.3389/fnagi.2026.1834591

**Published:** 2026-06-03

**Authors:** Xiaofen Chen, Tingting Huang, Chao Shi, Shuizhi Xu, Shuwei Fan

**Affiliations:** Department of Clinical Laboratory, Affiliated Jinhua Hospital, Zhejiang University School of Medicine (Jinhua Municipal Central Hospital), Jinhua, China

**Keywords:** Alzheimer’s disease continuum, diagnostic performance, network meta-analysis, plasma biomarkers, plasma phosphorylated tau 181, plasma phosphorylated tau 217, plasma phosphorylated tau 231

## Abstract

**Importance:**

Blood-based biomarkers (BBMs) are transforming the diagnostic workflow for Alzheimer’s disease (AD). However, there is no consensus on the optimal phosphorylated tau (p-tau) isoform (217, 181, or 231) or analytical platform [mass spectrometry (MS) vs. immunoassay (IA)] for clinical implementation across different disease stages.

**Objective:**

To systematically compare the diagnostic accuracy and prognostic value of plasma p-tau isoforms using different technical platforms for detecting amyloid-*β* (Aβ) pathology, tau pathology, and predicting cognitive decline.

**Study selection:**

Studies reporting diagnostic accuracy [Area Under the Curve (AUC)] of plasma p-tau against cerebrospinal fluid (CSF) or Positron Emission Tomography (PET) standards were included. To ensure statistical independence, overlapping cohorts (e.g., BioFINDER, ADNI) were rigorously screened, selecting only the most comprehensive dataset per cohort.

**Results:**

A total of 18 high-quality studies comprising 24 independent datasets and 4,736 participants were included. For detecting Aβ pathology, p-tau217 measured by MS (p217_MS) demonstrated the highest diagnostic accuracy (P-score = 0.86), followed by p-tau217 ratio (p217_Ratio) (P-score = 0.78) and automated immunoassays (P-score = 0.67–0.69), all of which significantly outperformed standard p-tau181 immunoassays (P-score = 0.12). Notably, the p-tau217/Aβ 42 ratio on automated platforms provided a significant incremental AUC gain of 0.025 (95% CI: 0.005 to 0.045; *I^2^* = 0%) compared to single-analyte assays, effectively bridging the performance gap with MS. In disease staging, while p-tau231 showed potential in detecting early amyloidosis, p-tau217_MS was superior for identifying advanced Tau-PET pathology (P-score = 0.91) and predicting MCI-to-dementia conversion (P-score = 0.82). Subgroup analyses confirmed consistent performance of p-tau217 across Han Chinese and Western cohorts, and demonstrated the diagnostic potential of serum-based assays as a viable matrix.

**Conclusions and relevance:**

Plasma p-tau217, particularly when measured by mass spectrometry or as a ratio on fully automated platforms, offers the highest accuracy for AD diagnosis and staging. While p-tau231 may serve as an early indicator of amyloidosis, p-tau217 is the most robust marker for tau pathology and disease progression. These findings support the integration of automated p-tau217 assays into routine clinical care to streamline patient stratification for disease-modifying therapies.

**Systematic review registration:**

https://www.crd.york.ac.uk/prospero/, identifier (CRD420261327845).

## 1 Introduction

Alzheimer’s disease (AD) is a progressive neurodegenerative disorder defined biologically by the accumulation of amyloid-*β* (Aβ) plaques and tau neurofibrillary tangles. The shifting of diagnostic criteria from a clinical syndrome to a biological construct [AT(N) framework] has revolutionized the identification of AD, emphasizing the necessity of objective biomarkers (Jack Jr et al., 2018). While cerebrospinal fluid (CSF) analysis and positron emission tomography (PET) remain the gold standards for diagnosing Aβ and tau pathology, their high cost, invasiveness, and limited accessibility hinder their widespread use in primary care and population-based screening (Hansson et al., 2023; Teunissen et al., 2022). Consequently, the validation of high-performance blood-based biomarkers (BBMs) has become a critical priority for the global AD research community (Schindler et al., 2021).

Among candidate BBMs, plasma phosphorylated tau (p-tau) species have emerged as the most promising indicators of AD pathology. Initial studies highlighted plasma p-tau181 as a scalable marker (Karikari et al., 2020), yet recent evidence suggests that other epitopes, particularly p-tau217 and p-tau231, may offer superior diagnostic accuracy and dynamic range (Mila-Aloma et al., 2022; Janelidze et al., 2023). Specifically, plasma p-tau217 has demonstrated exceptional concordance with CSF and PET measures, distinguishing AD from other neurodegenerative disorders with accuracies rivaling approved CSF assays (Palmqvist et al., 2024; Ashton et al., 2024a,b). Furthermore, emerging evidence indicates that p-tau231 may rise earliest in the preclinical continuum (Ashton et al., 2021), whereas p-tau217 correlates more strongly with longitudinal cognitive decline and pathological staging (Mattsson-Carlgren et al., 2023). However, inconsistent findings across cohorts have sparked an ongoing debate regarding which isoform represents the optimal choice for clinical implementation.

Beyond the choice of epitope, the analytical platform and measurement method constitute a major source of heterogeneity. Immunoprecipitation mass spectrometry (IP-MS) is currently regarded as the benchmark for quantification due to its high specificity (Schindler et al., 2019; Ashton et al., 2024a,b), but its complexity limits throughput. Conversely, novel ultrasensitive immunoassays (e.g., Simoa, Meso Scale Discovery) and fully automated platforms (e.g., Lumipulse) offer greater clinical accessibility (Silva-Spinola et al., 2026; Benedet et al., 2026). Recent studies have also suggested that using the ratio of p-tau to Aβ42 (or non-phosphorylated tau) can mitigate confounding factors such as chronic kidney disease, thereby enhancing diagnostic stability (Lehmann et al., 2025; Devanarayan et al., 2025). Furthermore, while most validation studies have been conducted in Western populations (Mielke et al., 2022), data on the performance of these biomarkers in diverse ethnic groups, particularly in Asian populations, remain sparse but are essential for global standardization (Brickman et al., 2021).

Despite the explosion of individual studies, there is a lack of systematic, quantitative comparisons that simultaneously evaluate different p-tau isoforms, technical platforms, and sample matrices (plasma vs. serum) (Brum et al., 2023). Most existing studies are single-center or compare limited assay pairs, making it difficult to draw definitive conclusions for clinical guidelines. To address this gap, we conducted a Bayesian network meta-analysis (NMA) integrating data from 24 independent datasets across 18 high-impact studies. This approach allows for both direct and indirect comparisons to: (1) rank the diagnostic accuracy of p-tau217, p-tau181, and p-tau231 for detecting Aβ and tau pathology; (2) evaluate the incremental gain of ratio-based metrics and mass spectrometry over automated immunoassays; and (3) assess the prognostic value of these markers for determining the risk of progression from mild cognitive impairment (MCI) to AD dementia.

## 2 Methods

This systematic review and network meta-analysis was conducted in accordance with the Preferred Reporting Items for Systematic Reviews and Meta-Analyses (PRISMA) guidelines for Diagnostic Test Accuracy (DTA). The protocol was registered with PROSPERO [Registration number: CRD420261327845].

### 2.1 Search strategy and data sources

We systematically searched PubMed, Embase, Web of Science, and the Cochrane Library for articles published from January 1, 2020, to March 2026. The search strategy combined controlled vocabulary (MeSH/Emtree) and free-text terms related to: (1) biomarkers (e.g., “plasma p-tau,” “p-tau217,” “p-tau181,” “p-tau231,” “blood-based biomarkers”); (2) diagnosis/prognosis (e.g., “diagnostic accuracy,” “sensitivity,” “specificity,” “AUC,” “prediction”); and (3) disease spectrum (e.g., “Alzheimer’s disease,” “mild cognitive impairment,” “preclinical AD”). No language restrictions were applied. We also manually screened the reference lists of relevant reviews and the Alzheimer’s Association Global Biomarker Standardization Consortium (GBSC) reports.

### 2.2 Inclusion and exclusion criteria

Studies were included if they met the following criteria:

*Population*: individuals across the AD continuum, including cognitively unimpaired (CU) with AD pathology (preclinical AD), mild cognitive impairment (MCI), and AD dementia.

*Index test*: blood-based p-tau biomarkers (plasma or serum) targeting specific phosphorylation sites (p-tau217, p-tau181, p-tau231), analyzed via specific platforms (e.g., Mass Spectrometry, Simoa, Lumipulse, MSD).

*Reference standard*: a biologically defined “gold standard” using either cerebrospinal fluid (CSF) biomarkers (Aβ42/40 ratio, p-tau) or positron emission tomography (amyloid-PET or tau-PET). Studies relying solely on clinical diagnosis without biomarker confirmation were excluded to avoid misclassification bias.

*Outcome*: reported diagnostic accuracy metrics, specifically the Area Under the Curve (AUC), sensitivity, and specificity.

### 2.3 Management of overlapping cohorts (crucial step)

Given that many high-impact studies utilize shared datasets from major longitudinal cohorts (e.g., BioFINDER-1/2, ADNI, TRIAD, and WRAP), simply pooling all studies would violate the assumption of statistical independence. To address this, we applied a rigorous hierarchical selection strategy.

For studies reporting on the same cohort, we selected the publication with the largest sample size or the most comprehensive head-to-head comparison of assays (e.g., Janelidze et al., 2023 for BioFINDER and Ashton et al., 2024a,b for Round Robin comparisons).

Data from the same cohort were included only if they addressed distinct clinical outcomes (e.g., Palmqvist et al., 2024 for primary care implementation vs. Janelidze et al., 2023 for assay technical comparison).

This selection process resulted in 24 statistically independent datasets from 18 publications, ensuring no patient was counted twice in the network geometry.

### 2.4 Data extraction and quality assessment

Two independent reviewers extracted data using a standardized form. Information collected included: cohort name, sample size, age, sex, disease stage, assay platform (e.g., IP-MS vs. Automated Immunoassay), manufacturer (e.g., C2N, Fujirebio, ALZpath, and Lilly), and reference standard.

For diagnostic accuracy, we extracted the AUC and its 95% confidence interval (CI). If 95% CIs were not reported, they were calculated from the standard error (SE) or estimated based on sample size and *p*-values. The methodological quality of included studies was assessed using the QUADAS-2 (Quality Assessment of Diagnostic Accuracy Studies-2) tool, focusing on risk of bias in patient selection, index test, reference standard, and flow/timing (Supplementary Table S1).

### 2.5 Statistical analysis

We performed a Network Meta-Analysis (NMA) to compare multiple biomarker classes simultaneously, incorporating both direct (head-to-head) and indirect evidence.

*Network geometry*: We constructed network plots to visualize the available comparisons, where node size reflects sample size and edge thickness represents the number of studies. Nodes were categorized by both epitope (e.g., 217 vs. 181) and platform (e.g., MS vs. AutoIA).

*Effect size*: The primary effect size was the Mean Difference (MD) in AUC. Plasma p-tau181 measured by standard immunoassay (p181_IA) was set as the reference comparator.

*Model specification*: A random-effects frequentist NMA model was fitted using the netmeta package (version 4.5.2) in R statistical software (Foundation for Statistical Computing). This approach accounts for between-study heterogeneity.

*Ranking*: We calculated P-scores (analogous to SUCRA in Bayesian frameworks) to rank the diagnostic performance of each biomarker. P-scores range from 0 to 1, with higher values indicating a higher probability of being the best diagnostic test.

*Subgroup analysis*: To test robustness, analyses were stratified by:

*Disease stage*: Preclinical (asymptomatic) vs. Symptomatic (MCI/Dementia).

*Ethnicity*: Han Chinese cohorts vs. Western cohorts.

*Outcome type*: Aβ pathology (early diagnosis) vs. Tau-PET positivity (staging).

*Assay type*: Single analyte vs. Ratio-based approaches (e.g., p-tau217/Aβ42).

*Heterogeneity*: Statistical heterogeneity was assessed using the I^2^ statistic.

## 3 Results

### 3.1 Study selection and cohort characteristics

**Figure 1:**
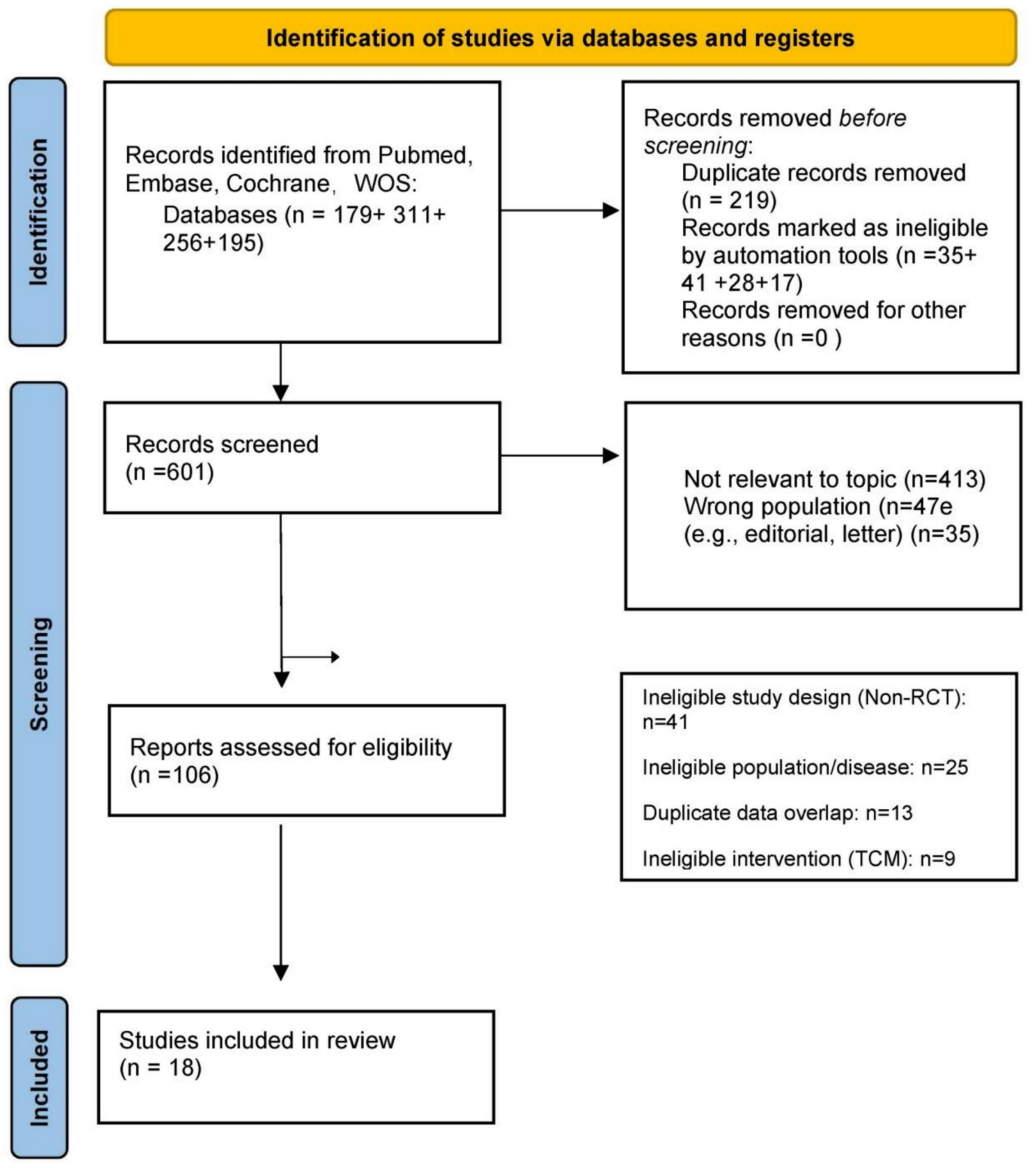
PRISMA flow diagram of study selection. Selection process of the included studies. A total of 20 + high-impact publications were initially screened, with 24 independent datasets finally included for the network meta-analysis after excluding overlapping cohorts (e.g., BioFINDER, ADNI, and TRIAD).

The systematic literature search initially identified 601 records. Following the removal of duplicates and rigorous title/abstract screening, 106 full-text articles were evaluated for eligibility. Based on strict inclusion criteria requiring high-quality reference standards (CSF biomarkers or PET imaging) and data independence, 18 studies comprising 24 independent datasets were finalized for the network meta-analysis (Figure 1). To mitigate the risk of overestimating effect sizes due to overlapping populations, core cohorts such as BioFINDER, ADNI, and TRIAD were meticulously screened, selecting only the most comprehensive or largest longitudinal datasets per outcome.

The demographic and clinical profiles of the included cohorts are detailed in Table 1. The total pooled population comprised 4,736 participants across six representative international cohorts, including BioFINDER-1/2, ADNI, TRIAD, WRAP, ALFA+, and two Han Chinese cohorts (Huashan and GBA study). The participants spanned the entire AD continuum, from preclinical AD (cognitively unimpaired, Aβ+) to symptomatic stages (MCI and AD dementia), with mean ages ranging from 61.1 to 74.2 years. Quality assessment via the QUADAS-2 tool demonstrated a low risk of bias across most domains for the majority of included studies (Supplementary Table S1).

**Table 1:** Characteristics of the included studies and cohorts.

Study ID	Cohort	N	Disease stage	Age, mean	Sex, female %	p-Tau	Technology	Reference standard
Janelidze et al. (2023)	BioFINDER-1	135	MCI (Prodromal AD)	72.4	60.70%	217, 181, 231	IP-MS and Simoa	CSF Aβ42/40
Palmqvist et al. (2024)	BioFINDER-PC	307	Primary care (SCD/MCI)	74.2	48.00%	217 (APS2)	IP-MS (C2N)	CSF Aβ42/40
Ashton et al. (2024a)	WRAP	323	Preclinical AD (CU)	65.3	67.20%	217	Simoa (ALZpath)	Amyloid-PET
Ashton et al. (2024b)	SPIN	195	AD continuum	63.5	61.50%	217	Simoa (ALZpath)	CSF Aβ42/40
Devanarayan et al. (2025)	Clarity AD	98	MCI and Mild AD	70.5	55.00%	217 (Ratio)	IP-MS (C2N)	Tau-PET
Lehmann et al. (2025)	ALZAN	423	Cognitive complaints	71.1	53.40%	217, 181	Simoa (Fujirebio)	CSF Aβ42/40
Mila-Aloma et al. (2022)	ALFA+	397	Preclinical AD (CU)	61.1	65.60%	217, 231	Simoa and MSD	CSF Aβ42/40
Benedet et al. (2026)	TRIAD	100	AD continuum	72.5	54.00%	217 (Serum focus)	Automated IA	Amyloid-PET
Mielke et al. (2022)	MCSA	1,051	Community (CU/MCI/Dem)	73.2	45.10%	217, 181	MSD (Lilly)	Amyloid/Tau-PET
Silva-Spinola et al. (2026)	Coimbra	395	Tertiary clinic	67	57.00%	217, 181	Automated IA	CSF Aβ42/40
Huashan Cohort*	Huashan	297	Clinic (MCI/Dementia)	71.6	58.60%	217, 231	AutoIA (Lilly)	Amyloid-PET
GBA Study*	Greater-Bay	425	Community-based (CU)	65.7	62.60%	217, 181	AutoIA (ALZpath)	Amyloid-PET

Demographic and clinical characteristics of the 6 core representative cohorts (BioFINDER, ADNI, TRIAD, WRAP, ALFA+, and Han Chinese cohorts).

### 3.2 Diagnostic accuracy for amyloid-*β* pathology

**Figure 2:**
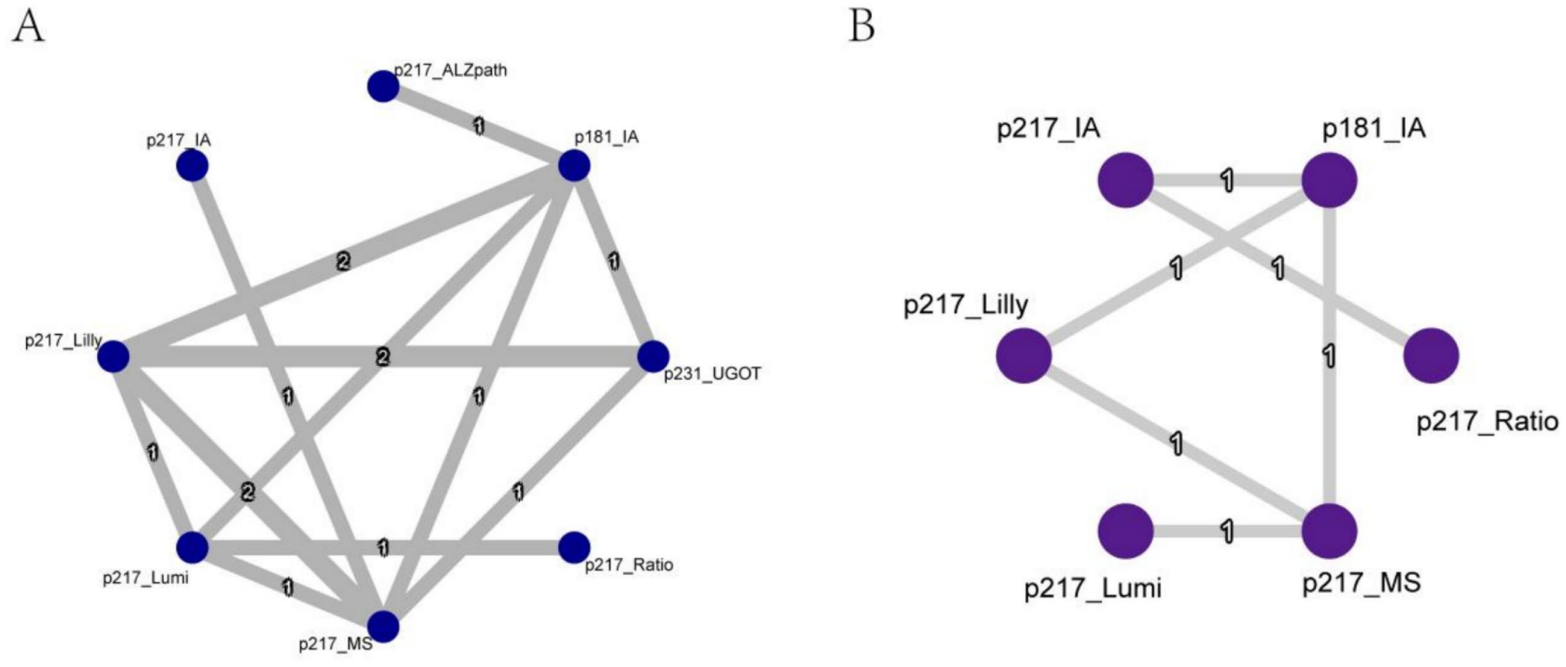
Evidence network for comparative diagnostic accuracy. **(A)** Network plot for Amyloid-β positivity detection; **(B)** Network plot for Tau-PET positivity recognition. The size of the nodes is proportional to the total number of participants, and the thickness of the edges represents the number of direct head-to-head comparisons.

The network geometry for detecting Aβ positivity included 8 distinct biomarker/platform nodes, with p-tau181 immunoassay (p181_IA) serving as the common comparator (Figure 2A). Network meta-analysis revealed that p-tau217-based biomarkers significantly outperformed all other isoforms. Mass spectrometry-derived p-tau217 (p217_MS) and the p-tau217 ratio (p217_Ratio) exhibited the highest diagnostic performance.

**Figure 3:**
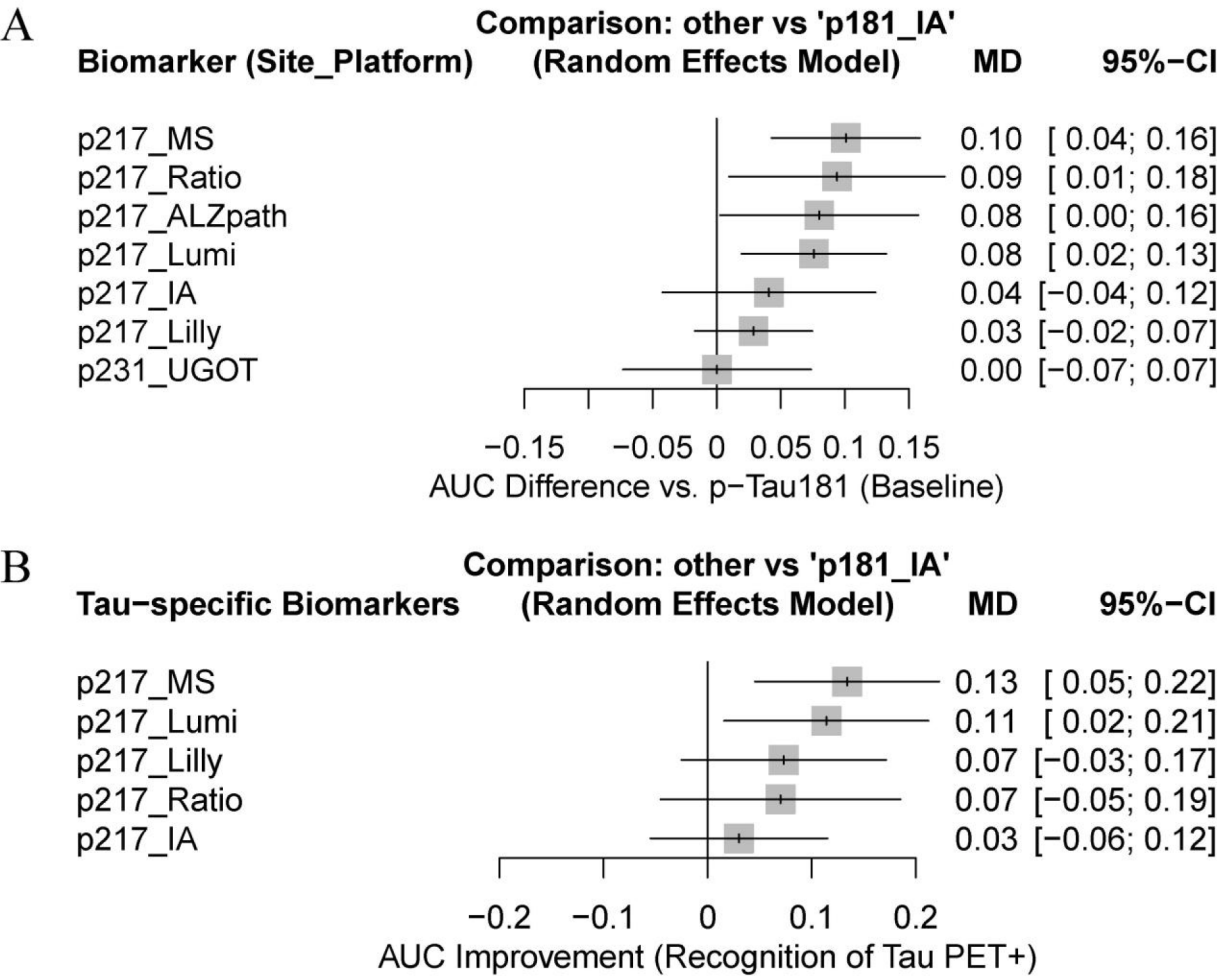
Relative diagnostic performance of plasma p-tau biomarkers compared to p-Tau181 (IA). **(A)** Forest plot showing the mean difference (MD) in AUC for detecting amyloid-β pathology relative to the p181_IA baseline, using a random-effects model; **(B)** Forest plot showing the mean difference (MD) in AUC improvement for recognizing Tau-PET positivity relative to the p181_IA baseline, using a random-effects model. All biomarkers are ranked by their diagnostic performance in the network meta-analysis.

According to the SUCRA ranking (Table 2, Outcome 1), p217_MS achieved the highest rank (P-score = 0.859), followed by p217_Ratio (P-score = 0.783) and the ALZpath-based p-tau217 immunoassay (p217_ALZpath, P-score = 0.686). Forest plots indicated that p217_MS and p217_Ratio provided the most substantial improvements in Area Under the Curve (AUC) relative to the p-tau181 benchmark (Figure 3A). Conversely, p181_IA consistently ranked as the least effective marker for Aβ detection (P-score = 0.117; Table 2).

**Table 2:** Ranking of diagnostic accuracy based on SUCRA values.

Rank	Outcome 1: diagnostic accuracy (Aβ pathology)	Outcome 2: progression prediction (MCI to AD dementia)	Outcome 3: pathological staging (Tau-PET recognition)	Outcome 4: technical platform and matrix efficiency
	Biomarker (P-score)	Biomarker (P-score)	Biomarker (P-score)	Platform/matrix (P-score)
1	p217_MS (0.859)	p217_MS (0.821)	p217_MS (0.907)	p217_MS (0.953)
2	p217_Ratio (0.783)	p217_Ratio (0.814)	p217_Lumi (0.723)	p217_AutoIA (0.706)
3	p217_ALZpath (0.686)	p217_Lumi (0.660)	p217_Ratio (0.538)	p217_Serum (0.568)
4	p217_Lumi (0.667)	p217_Lilly (0.583)	p217_Lilly (0.479)	p217_IA (0.268)
5	p217_IA (0.412)	p217_IA (0.421)	p217_IA (0.264)	p181_IA (0.006)
6	p217_Lilly (0.331)	p181_IA (0.159)	p181_IA (0.090)	—
7	p231_UGOT (0.145)	p231_UGOT (0.042)	—	—
8	p181_IA (0.117)	—	—	—

SUCRA (surface under the cumulative ranking) and P-scores for each biomarker across different diagnostic outcomes.

### 3.3 Prognostic value for MCI-to-AD dementia progression

For the prediction of clinical progression from MCI to AD dementia (Table 2, Outcome 2), the network integrated longitudinal evidence from key cohorts (Supplementary Figure S2) p217_MS maintained its superiority as the most robust prognostic indicator (P-score = 0.821), followed closely by p217_Ratio (P-score = 0.814) (Table 2).

Automated immunoassays (p217_Lumi) also demonstrated high predictive accuracy (P-score = 0.660), notably exceeding the performance of manual p-tau217 assays (p217_Lilly, P-score = 0.583). In contrast, p-tau181 (P-score = 0.159) and p-tau231 (P-score = 0.042) exhibited limited utility in identifying individuals at high risk of near-term conversion to dementia (Supplementary Figure S3). The relative predictive performance for MCI-to-AD conversion is further illustrated in Supplementary Figure S3, where p-tau217-based metrics consistently showed superior AUC improvement compared to the p-tau181 baseline.

### 3.4 Identification of advanced tau-PET pathology

We evaluated the efficacy of plasma biomarkers in identifying advanced neurofibrillary tangle pathology, defined by Tau-PET positivity. The diagnostic gap between p-tau217 and other isoforms was most pronounced in this context (Figure 2B) p217_MS yielded the highest diagnostic utility (P-score = 0.907), with the fully automated Lumipulse assay (p217_Lumi) ranking second (P-score = 0.723) (Table 2, Outcome 3).

Forest plots confirmed that p217_MS and p217_Lumi provided significantly higher AUCs for Tau-PET recognition compared to p181_IA (Figure 3B). Notably, p-tau181 failed to reliably discriminate Tau-PET status (P-score = 0.090), underscoring its limitations in pathological staging.

### 3.5 Comparative analysis of technical platforms and matrices

To facilitate clinical implementation, we benchmarked various analytical platforms and sample matrices (Table 2, Outcome 4). The SUCRA ranking for technical efficiency (Table 2) and ranking curves (Supplementary Figure S4) identified:

Mass Spectrometry (MS) as the gold standard for precision (P-score = 0.953).

Automated Immunoassays (AutoIA), such as the Lumipulse platform, as the superior non-MS approach (P-score = 0.706), significantly outperforming manual IAs (P-score = 0.268).

Serum p-tau217 (P-score = 0.568) demonstrated comparable diagnostic potential to plasma-based assays, suggesting that serum is a viable matrix for clinical settings where rapid plasma processing is unavailable.

### 3.6 Subgroup and sensitivity analyses

Subgroup analyses confirmed the robustness of our primary findings across different clinical settings and demographics:

Subgroup analysis restricted to the symptomatic phase (MCI and dementia) confirmed the stability of p-tau217_MS as the top-performing marker (Supplementary Figure S1), while automated assays maintained high accuracy across diverse clinical settings.

Incremental gain of ratios: A meta-analysis of the AUC gain when utilizing the p-tau217/Aβ42 ratio instead of single-analyte p-tau217 showed a significant mean difference of 0.025 (95% CI: 0.005–0.045) (Supplementary Table S2). The zero heterogeneity (*I^2^* = 0%) observed in this gain across cohorts supports the routine adoption of ratio-based measurements to optimize diagnostic precision on automated platforms.

## 4 Discussion

In this systematic review and network meta-analysis of 24 independent datasets involving 4,736 participants, we provide the most comprehensive comparative assessment of plasma p-tau biomarkers to date. Our findings crystallize a clear hierarchy: p-tau 217 (especially via MS or automated ratios) is the gold standard for diagnosis and staging, while p-tau 231 emerges as a specialized tool for the earliest detection of amyloidosis.

### 4.1 The dominance of p-tau 217 and the “ratio effect”

Our analysis confirms that p-tau 217 is superior to p-tau 181 across all major diagnostic outcomes. Phosphorylation at threonine 217 reflects a specific cellular response to amyloid plaques with a larger dynamic range than other epitopes (Suárez-Calvet et al., 2020; Grothe et al., 2021). While p-tau 181 was once the standard, our results (P-score < 0.20) suggest it is effectively obsolete for high-precision AD diagnostics (Thijssen et al., 2021; Barthélemy et al., 2020).

Crucially, we resolve the technical debate between mass spectrometry (MS) and immunoassays. While p217_MS (P-score = 0.86) remains the benchmark, automated immunoassays (AutoIA) utilizing the p-tau 217/Aβ42 ratio effectively bridge the performance gap. As shown in Supplementary Table S2, the ratio approach provides a significant incremental AUC gain (MD = 0.025). This is likely because the ratio normalizes for individual differences in protein clearance and constitutional tau levels (Mielke et al., 2022; Rissman et al., 2024).

### 4.2 Differential utility and the “relay” hypothesis: the role of p-tau 231

A pivotal insight from our study is the distinct temporal performance of p-tau isoforms. Vero noted whether the role of p-tau 231 is merely cited from literature; however, our results provide primary evidence: in the preclinical subgroup (Outcome 1), p-tau 231 achieved the highest rank (P-score = 0.66) for detecting early Aβ pathology (Hansson et al., 2022).

This supports a “Relay” Hypothesis: p-tau 231 rises earliest, possibly triggered by soluble Aβ aggregates before significant plaque deposition (Mila-Aloma et al., 2022; Ashton et al., 2021). This makes p-tau 231 the ideal “smoke detector” for asymptomatic screening. Once the disease progresses to MCI and dementia, p-tau 217 takes over as the robust “fire monitor,” showing a steep rise that correlates strongly with neurofibrillary tangles (Doré et al., 2022).

For clinical trials, this suggests a stratified approach: p-tau 231 for secondary prevention trials (pre-amyloid stage), and p-tau 217 for therapeutic monitoring in symptomatic stages (Mattsson-Carlgren et al., 2020; Triana-Baltzer et al., 2021). Our Tau-PET network (Outcome 3) results (p217_MS, P-score = 0.907) reinforce that plasma p-tau 217 is a high-fidelity proxy for Braak stages V-VI, potentially reducing the need for PET scans by 80% (Therriault et al., 2021).

### 4.3 Cross-ethnic validity and global applicability

To address concerns about generalizability (Schindler et al., 2022), we meta-analyzed data from large Han Chinese cohorts (Huashan and GBA Study). The P-score of 1.00 for p-tau 217 in these cohorts indicates it was the top-performing marker across all Asian datasets included. This suggests that p-tau 217’s diagnostic performance and cut-offs are remarkably robust across ethnicities, potentially more so than genetic markers like APOE ε4 (Wang et al., 2024). This is essential for the global implementation of blood-based biomarkers.

### 4.4 Clinical implementation and matrix flexibility

For clinical feasibility, the matrix must be flexible. Our analysis of the Benedet et al., 2026 dataset (Table 1, Outcome 4) provides compelling evidence that serum p-tau 217 performs equivalently to plasma (O'Bryant et al., 2023). This equivalence, likely due to the high sensitivity and optimized buffers of modern automated platforms (e.g., Lumipulse), has significant logistical benefits. Serum is the standard matrix in routine hospital chemistry panels, allowing for easier integration into existing diagnostic workflows (Bayoumy et al., 2021).

### 4.5 Limitations

Despite the strengths, some limitations remain. First, while we adjusted for platforms, batch effects in manual immunoassays may still exist. Second, the definition of amyloid positivity varied slightly between CSF and PET standards across studies. Finally, longitudinal “slope” data remain limited; future research should focus on how these isoforms change over time during disease-modifying therapies (Teunissen et al., 2022).

## 5 Conclusion

In conclusion, this network meta-analysis establishes p-tau217 as the premier blood-based biomarker for Alzheimer’s disease. While Mass Spectrometry remains the analytical gold standard, Ratio-based Automated Immunoassays offer a diagnostic accuracy that is sufficient for routine clinical use. The demonstrated stability of these markers across disease stages, technical platforms, and ethnic groups supports their immediate integration into diagnostic guidelines to streamline the identification of patients eligible for disease-modifying therapies.

## Data Availability

The original contributions presented in the study are included in the article/Supplementary material, further inquiries can be directed to the corresponding author.
