# Supplementary material for: Diagnostic performance of plasma p-Tau217, p-Tau181, and p-Tau231 across the Alzheimer’s disease continuum: a network meta-analysis
Source: Front Aging Neurosci. 2026 Jun 3;18:1834591. doi: 10.3389/fnagi.2026.1834591 (PMC13272307; doi:10.3389/fnagi.2026.1834591)
Supplement: Supplementary file 1 [file Data_Sheet_1.pdf]

Study_ID	Year	PMID_DOI	Country
Devanarayan_2025_VS1	2025	10.1002/alz.14411	USA
Devanarayan_2025_VS2	2025	10.1002/alz.14411	USA
Brickman_2021_Autopsy	2021	10.1002/alz.12301	USA
Brickman_2021_Clinical	2021	10.1002/alz.12301	USA
Mila-Aloma_2022	2022	10.1038/s41591-022-01925-w	Spain
Devanarayan_2025_Comb	2025	10.1002/alz.70881	USA
Yaari_2025_Baseline	2025	10.1002/alz.70662	USA/Japan
Palmqvist_2024	2024	10.1001/jama.2024.13855	Sweden
Janelidze_2023	2023	10.1093/brain/awac333	Sweden
Devanarayan_2025	2025	10.1002/alz.14411	USA
Ashton_2024	2024	10.1001/jamaneurol.2023.5319	Multi-natior
Lehmann_2025	2025	10.1016/j.ebiom.2025.105805	France
Mila-Aloma_2022	2022	10.1038/s41591-022-01925-w	Spain
Brickman_2021	2021	10.1002/alz.12301	USA
Benedet_2026	2026	10.1093/clinchem/hvaf162	Canada
Janelidze_2023	2023	10.1093/brain/awac333	Sweden
Palmqvist_2024	2024	10.1001/jama.2024.13855	Sweden
Benedet_2026	2026	10.1093/clinchem/hvaf162	Canada
Ashton_2024	2024	10.1001/jamaneurol.2023.5319	USA
Lehmann_2025	2025	10.1016/j.ebiom.2025.105805	France
Silva-Spinola_2026	2026	10.1038/s41598-025-34241-7	Portugal

Cohort_Name	Sample_Size_Total	Population_Type	Disease_Stage
Clarity AD	98	Early AD (Aβ+)	MCI & Mild AD
ADNI	47	Preclinical/Early A	CU & MCI (Aβ+)
WHICAP	113	Community Study	Autopsy confirmed AD
WHICAP	297	Community Study	Clinical AD
ALFA+	397	Preclinical AD	CU
Clarity AD	57	Early AD (Aβ+)	MCI & Mild AD
TRAILBLAZER 3	2196	Preclinical AD	CU (Aβ+)
BioFINDER	1213	Primary/Secondary C	CU, MCI, Dementia
BioFINDER	135	Clinic	MCI (Prodromal AD)
Clarity AD	98	Clinical Trial	MCI & Mild AD
WRAP/SPIN	786	Community	CU & Cognitive Impair.
ALZAN	423	Memory Clinic	Cognitive complaints
ALFA+	397	Community	Preclinical AD (CU)
WHICAP	113	Community	Autopsy-confirmed AD
TRIAD	100	Clinic	AD Continuum
BioFINDER	135	Clinic	MCI
BioFINDER PC	307	Primary Care	Cognitive symptoms
TRIAD	100	Clinic	AD continuum
WRAP	323	Community	Preclinical
ALZAN	423	Clinic	Cognitive complaints
Coimbra	395	Clinic	Cognitive complaints

Age_Mean	Sex_Female_Percent	Biomarker_Name	Assay_Platform	Manufacturer
70.5	55.00%	plasma pTau217 rat	IP-MS	C2N Diagnostics
72.2	51.10%	plasma pTau217 rat	IP-MS	C2N Diagnostics
88.63	61.00%	plasma p-tau217	MSD	Lilly/C2N
81.87	67.00%	plasma p-tau217	MSD	Lilly/C2N
61.1	65.60%	plasma p-tau217	MSD	NR
71.6	50.90%	pTau217R + CSF MTB	IP-MS/LC-MS	C2N Diagnostics
70.2	65.60%	plasma p-tau217	ECLIA	Eli Lilly
74.2	48.00%	p-tau217 ratio (AP	IP-MS	C2N Diagnostics
72.4	60.70%	p-tau217 (WashU)	IP-MS	WashU/C2N
70.5	55.00%	plasma pTau217 rat	IP-MS	C2N Diagnostics
66.3	64.10%	p-tau217 (ALZpath)	Simoa	ALZpath
71.1	53.40%	pTau217/Aβ42 rati	Simoa	Fujirebio
61.1	65.60%	plasma p-tau217	MSD	NR
88.6	61.00%	plasma p-tau217	MSD	Lilly/C2N
72.5	54.00%	serum p-tau217	Lumipulse	Fujirebio
72.4	60.70%	p-tau217 (WashU)	IP-MS	WashU
74.2	48.00%	APS2 (p-tau217%)	IP-MS	C2N Diagnostics
72.5	54.00%	serum p-tau217	Automated IA	Lumipulse
65.3	67.20%	p-tau217	Simoa	ALZpath
71.1	53.40%	pTau217/Aβ42 rati	Simoa	Fujirebio
67	57.00%	p-tau217	Automated IA	Lumipulse

Matrix	Reference_Standard	PET_Tracer	Cut_off_Value_Blood
Plasma	Tau PET (WCGM)	[18F]MK6240	Optimized
Plasma	Tau PET (WCGM)	Flortaucipir	Optimized
Plasma	Neuropathology	N/A	Median
Plasma	Clinical Diagnosis	N/A	Median
Plasma	CSF Aβ42/40	N/A	z-score > 2
Plasma/CSI	Tau PET (MTL)	[18F]MK6240	Combined
Plasma	Amyloid/Tau PET	FBP/FTP	NR
Plasma	CSF Aβ42/40 & p-tau217	N/A	Predefined
Plasma	CSF Aβ42/40	N/A	Optimized
Plasma	Tau PET (WCGM)	[18F]MK6240	Optimized
Plasma	Amyloid PET	NR	0.42 pg/mL
Plasma	CSF Aβ42/40	N/A	Optimized
Plasma	CSF Aβ42/40	N/A	z-score > 2
Plasma	Neuropathology	N/A	Median
Serum	Amyloid PET	NR	0.17 (Serum)
Plasma	CSF Aβ42/40	N/A	Optimized
Plasma	CSF Aβ42/40	N/A	Predefined
Serum	Amyloid PET	NR	0.17 pg/mL
Plasma	Amyloid PET	NR	0.42 pg/mL
Plasma	CSF Aβ42/40	N/A	Optimized
Plasma	CSF Aβ42/40	N/A	0.17 pg/mL

TP	FP
NR	NR
NR	NR
NR	NR
NR	NR
NR	NR
NR	NR
NR	NR
NR	NR
NR	NR
NR	NR
NR	NR
NR	NR
NR	NR
NR	NR
NR	NR
NR	NR
NR	NR
NR	NR
NR	NR
NR	NR
NR	NR

FN
NR
NR
NR
NR
NR
NR
NR
NR
NR
NR
NR
NR
NR
NR
NR
NR
NR
NR
NR
NR
NR

TN	Sensitivity	Specificity	AUC
NR	0.91	0.73	0.92
NR	0.46	0.78	0.74
NR	NR	NR	0.84
NR	NR	NR	0.63
NR	NR	NR	0.797
NR	NR	NR	0.939
NR	NR	NR	NR
NR	0.91	0.91	0.97
NR	0.94	0.91	0.947
NR	0.91	0.73	0.92
NR	0.95	0.75	0.93
NR	0.85	0.87	0.927
NR	NR	NR	0.797
NR	NR	NR	0.84
NR	0.86	0.84	0.97
NR	0.944	0.906	0.947
NR	0.91	0.91	0.97
NR	0.86	0.84	0.97
NR	0.953	0.745	0.93
NR	0.852	0.789	0.927
NR	0.92	0.79	0.9

AUC_95CI_Lower	AUC_95CI_Upper
0.86	0.97
0.59	0.88
0.75	0.92
0.57	0.7
0.751	0.842
NR	NR
NR	NR
0.95	0.99
0.907	0.987
0.86	0.97
0.9	0.97
0.9	0.954
0.751	0.842
0.75	0.92
0.95	1
0.907	0.987
0.95	0.99
0.95	1
0.9	0.97
0.9	0.954
0.87	0.94

Head_to_Head_Comparison
Yes
Yes
Yes
Yes
Yes
Yes
No
Yes
Yes
Yes
Yes
Yes
Yes
Yes
Yes
Yes
Yes
Yes
Yes
Yes
Yes

Comparison_Pairs	QUADAS_Selection	QUADAS_Index	QUADAS_Reference
pTau217 vs MRI/Clinical	Low	Low	Low
MK6240 vs Flortaucipir	Low	Low	Low
p-tau217 vs p-tau181	Low	Low	Low
p-tau217 vs NfL/GFAP	Low	Low	Low
p-tau217 vs p-tau231	Low	Low	Low
Single vs Combined	Low	Low	Low
N/A	Low	Low	Low
Blood vs Physician	Low	Low	Low
10 assays H2H	Low	Low	Low
p-tau217 vs MRI	Low	Low	Low
Plasma vs CSF	Low	Low	Low
Ratio vs Individual	Low	Low	Low
p-tau217 vs p-tau231	Low	Low	Low
Multi-ethnic	Low	Low	Low
Serum vs Plasma	Low	Low	Low
p-tau217 vs p-tau181	Low	Low	Low
Blood vs Physician	Low	Low	Low
Serum vs Plasma	Low	Low	Low
ALZpath vs CSF	Low	Low	Low
Ratio vs Single	Low	Low	Low
p-tau217 vs p-tau181	Low	Low	Low

QUADAS_Flow	Funding_Source
Low	Eisai Inc
Low	Eisai Inc
Low	NIH/Eli Lilly
Low	NIH/Eli Lilly
Low	BBRC/ERC
Low	Eisai Inc
Low	Eli Lilly
Low	NIH/Lilly
Low	Swedish Research C
Low	Eisai Inc
Low	NIH/BBRC
Low	Fondation Research
Low	BBRC/ERC
Low	NIH/Lilly
Low	Weston Brain Inst
Low	Swedish Research C
Low	NIH/Lilly
Low	Weston Brain Inst
Low	NIH/BBRC
Low	Fondation Alzheime
Low	NR

Comments
Validation Set 1 data
Validation Set 2 (ADNI)
Comparison with autopsy
Multi-ethnic cohort
AUC includes risk factors
Combined marker model
Trial baseline data only
Prospective validation
Head-to-head comparison
VS1 Validation Set
3-range approach used
Real-life cohort
Younger population
High ADNC vs others
Serum matrix focus
Highest AUC in study
Primary Care cohort data
Serum matrix focus
Preclinical population
Real-world clinical setting
Tertiary hospital setting
