# Supplementary figures and images for: Diagnostic performance of plasma p-Tau217, p-Tau181, and p-Tau231 across the Alzheimer’s disease continuum: a network meta-analysis
Source: Front Aging Neurosci. 2026 Jun 3;18:1834591. doi: 10.3389/fnagi.2026.1834591 (PMC13272307; doi:10.3389/fnagi.2026.1834591)

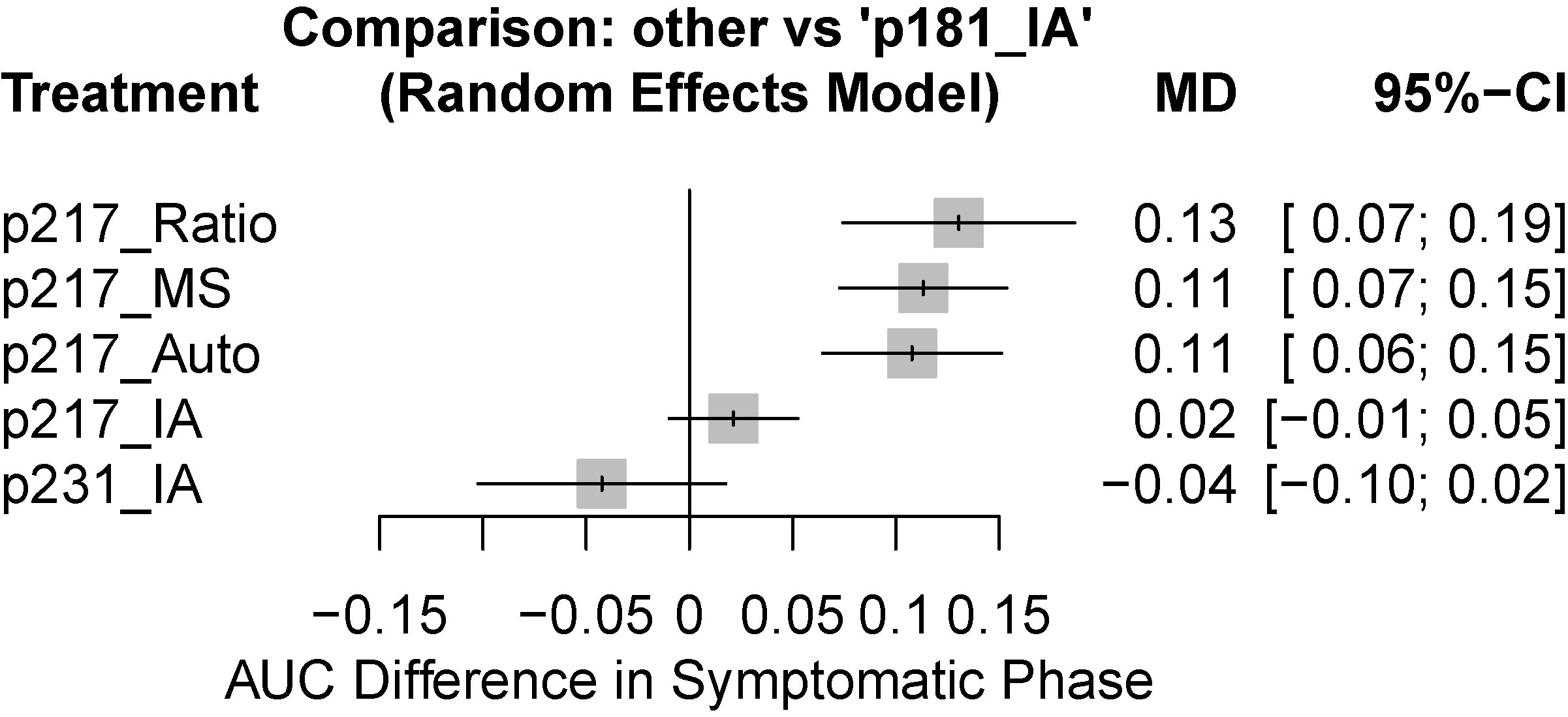

Supplement: Supplementary file 2 [file Image_1.tif]

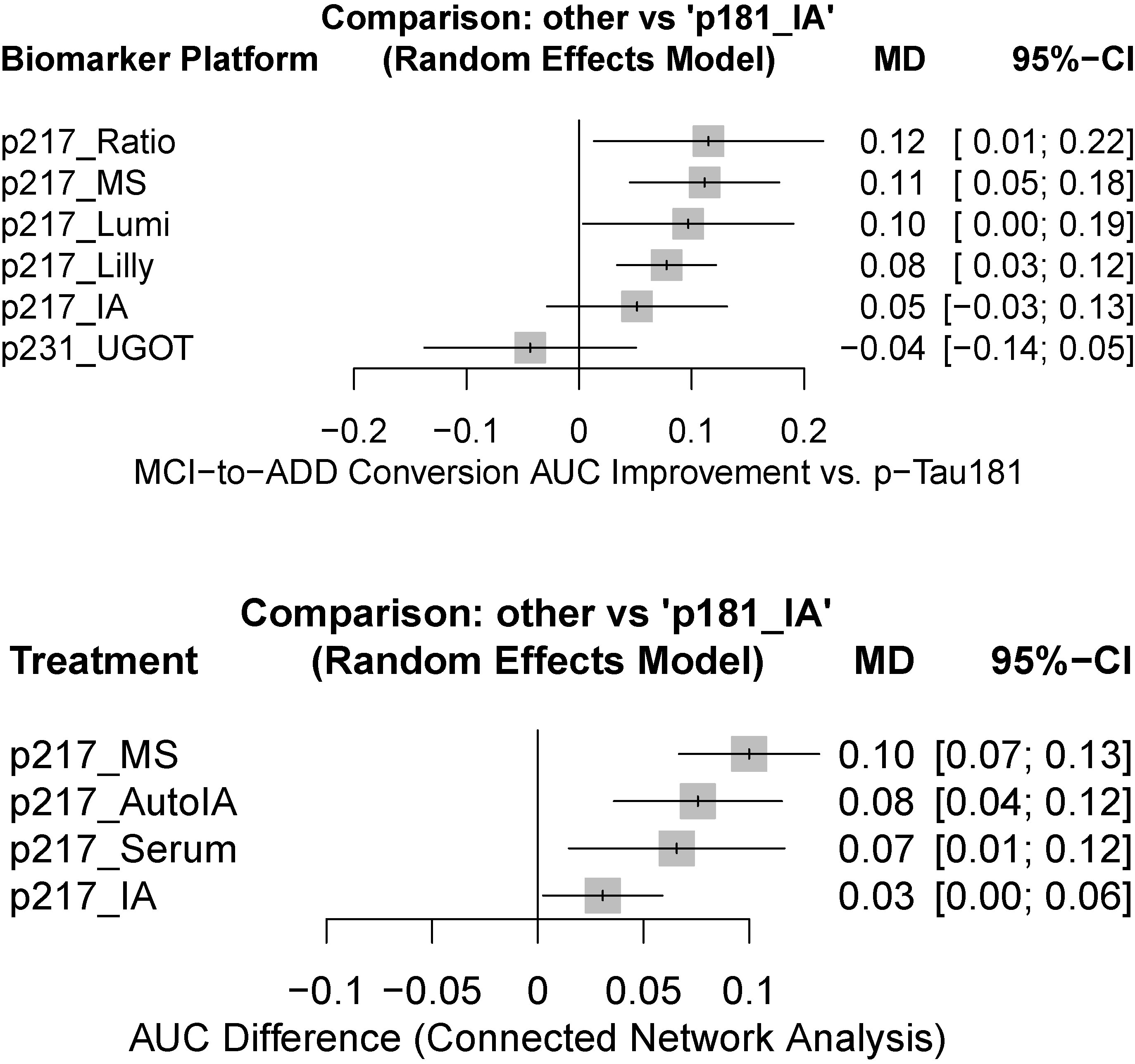

Supplement: Supplementary file 4 [file Image_3.tif]
