# Supplementary material for: Diagnostic performance of plasma p-Tau217, p-Tau181, and p-Tau231 across the Alzheimer’s disease continuum: a network meta-analysis
Source: Front Aging Neurosci. 2026 Jun 3;18:1834591. doi: 10.3389/fnagi.2026.1834591 (PMC13272307; doi:10.3389/fnagi.2026.1834591)
Supplement: Supplementary file 6 [file Table_1.docx]

| Study ID | Patient Selection | Index Test | Reference Standard | Flow and Timing | Overall Risk |
| --- | --- | --- | --- | --- | --- |
| Devanarayan_2025_VS1 | Low | Unclear | Low | Low | Low Risk |
| Devanarayan_2025_VS2 | Low | Low | Low | Low | Low Risk |
| Brickman_2021_Autopsy | Unclear | Low | Low | Low | Low Risk |
| Brickman_2021_Clinical | Low | Low | Low | Unclear | Low Risk |
| Mila-Aloma_2022 | Low | Low | Low | Low | Low Risk |
| Devanarayan_2025_Comb | Low | Unclear | Low | Low | Low Risk |
| Yaari_2025_Baseline | Low | Low | Low | Low | Low Risk |
| Palmqvist_2024 | Low | Low | Unclear | Low | Low Risk |
| Janelidze_2023 | Low | Low | Low | Low | Low Risk |
| Devanarayan_2025 | Unclear | Low | Low | Low | Low Risk |
| Ashton_2024 | Unclear | Low | Low | Low | Low Risk |
| Lehmann_2025 | Low | Low | Unclear | Low | Low Risk |
| Mila-Aloma_2022 | Low | Low | Low | Low | Low Risk |
| Brickman_2021 | Low | Low | Low | Unclear | Low Risk |
| Benedet_2026 | Low | Low | Low | Low | Low Risk |
| Janelidze_2023 | Low | Low | Unclear | Low | Low Risk |
| Palmqvist_2024 | Low | Low | Low | Low | Low Risk |
| Benedet_2026 | Low | Low | Low | Low | Low Risk |
| Ashton_2024 | Low | Unclear | Low | Unclear | Low Risk |
| Lehmann_2025 | Low | Low | Low | Low | Low Risk |
| Silva-Spinola_2026 | Unclear | Low | Low | Low | Low Risk |

Table S1. Risk of Bias Assessment (QUADAS-2).

Methodological quality assessment for each included study using the QUADAS-2 tool across four domains: patient selection, index test, reference standard, and flow/timing.
