# Supplementary material for: Diagnostic performance of plasma p-Tau217, p-Tau181, and p-Tau231 across the Alzheimer’s disease continuum: a network meta-analysis
Source: Front Aging Neurosci. 2026 Jun 3;18:1834591. doi: 10.3389/fnagi.2026.1834591 (PMC13272307; doi:10.3389/fnagi.2026.1834591)
Supplement: Supplementary file 7 [file Table_2.docx]

| Comparison Pair | Single AUC (Mean) | Ratio AUC (Mean) | MD [95% CI] | Heterogeneity (I²) | P-value |
| --- | --- | --- | --- | --- | --- |
| p217_Ratio vs. p217_AutoIA | 0.898 | 0.923 | 0.025 [0.005, 0.045] | 0% | <0.05 |
| p217_Ratio vs. p217_IA | 0.84 | 0.915 | 0.075 [0.032, 0.118] | 0% | <0.01 |

Table S2. Incremental Gain of Ratio-based vs. Single-analyte Biomarkers.

Meta-analysis of the AUC gain when using ratios (p217/Aβ42). The analysis shows zero heterogeneity (I² = 0%), supporting the robustness of the ratio approach.
