# Supplementary material for: Diagnostic performance of plasma p-Tau217, p-Tau181, and p-Tau231 across the Alzheimer’s disease continuum: a network meta-analysis
Source: Front Aging Neurosci. 2026 Jun 3;18:1834591. doi: 10.3389/fnagi.2026.1834591 (PMC13272307; doi:10.3389/fnagi.2026.1834591)
Supplement: Supplementary file 8 [file Table_3.docx]

Table S3. Node-splitting results for inconsistency assessment in the Tau-PET (Outcome 3) Network

| Comparison Pair (A vs. B) | Direct Evidence (MD in AUC) | Indirect Evidence (MD in AUC) | Network Estimate (Combined MD) | P-value (Inconsistency) |
| --- | --- | --- | --- | --- |
| p217_MS vs. p181_IA | 0.132 | 0.128 | 0.13 | 0.854 |
| p217_Lumi vs. p181_IA | 0.105 | 0.115 | 0.11 | 0.621 |
| p217_MS vs. p217_Lumi | 0.021 | 0.019 | 0.02 | 0.912 |
| p217_Ratio vs. p181_IA | 0.075 | 0.068 | 0.071 | 0.785 |
| p217_IA vs. p181_IA | 0.035 | 0.03 | 0.032 | 0.547 |
